# Burden of post-traumatic stress disorder acute exacerbations during the commemorations of the genocide against Tutsis in Rwanda: a cross-sectional study

**DOI:** 10.11604/pamj.2018.30.216.15663

**Published:** 2018-07-17

**Authors:** Jean Damascene Kabakambira, Gladys Uwera, Marthe Hategeka, Marie Louise Kayitesi, Célestin Kaputu Kalala Malu, Celestin Hategeka

**Affiliations:** 1 National Institutes of Health, Bethesda, MD, United States; 2 University Teaching Hospital of Kigali, Kigali, Rwanda; 3 School of Healthcare, Vancouver Career College, Burnaby, Canada; 4 University Teaching Hospital of Butare, Huye, Rwanda; 5 Centre for Health Services and Policy Research, School of Population and Public Health, Faculty of Medicine, University of British Columbia, Vancouver, BC, Canada; 6 Collaboration for Outcomes Research and Evaluation, Faculty of Pharmaceutical Sciences, University of British Columbia, Vancouver, BC, Canada

**Keywords:** Post-traumatic stress disorder, post-traumatic stress disorder acute exacerbation, mental health, genocide, Rwanda

## Abstract

**Introduction:**

Following the 1994 genocide against Tutsis in Rwanda, the prevalence of post-traumatic stress disorder (PTSD) is high. In a period of seven days every year in April, Rwandans gather to mourn the victims of the genocide. During this commemoration period, survivors living with chronic PTSD experience PTSD acute exacerbations (PAE). We assessed factors associated with severe PAE during the annual commemoration period of the genocide against Tutsis in Rwanda.

**Methods:**

We carried out a retrospective cross-sectional study that included people who had PAE during the commemoration week in April 2011 across Huye District in Rwanda. Our outcome measure was PAE categorized into three levels: < 15 minutes, 15-30 minutes, and > 30 minutes. Ordinal logistic regression analyses were performed to identify factors associated with severe PAE.

**Results:**

We enrolled 383 people with PAE, of whom 71.8% were female and 53.5% were aged 20-45 years. All participants reported history of PAE, of which 59.8% had experienced more than two PAE during the previous commemoration periods. 33.2% had PAE that lasted > 30 minutes. History of PAE (> twice) (OR = 1.86; 95% CI = 1.27-2.75) and having lost a partner in genocide (OR = 2.19; 95% CI = 1.01-4.81) were associated with severe PAE, after adjusting for sex and age.

**Conclusion:**

Our findings suggest that PAE is frequent during the commemoration periods. People who reported having more prior PAE and being widow (er) were more likely to have severe PAE. While history of PAE and bereavement status are non-modifiable factors, our findings could help identify and target these people who are at risk for severe PAE.

## Introduction

Mental health disorders remain a significant global health challenge, accounting for nearly 23% of global years lived with disability [1]. Posttraumatic stress disorder (PTSD) affects (post) conflict regions disproportionally [2,3]. PTSD can be defined as a common anxiety disorder that develops after exposure to a terrifying event or ordeal in which grave physical harm occurred or was threatened [4]. Numerous studies have established the frequent occurrence of PTSD among individuals exposed to traumas including wars, natural disasters, terrorist attacks, road traffic accidents and interpersonal violence [5-7]. Longitudinal studies indicate that PTSD symptoms appear shortly after the traumatic event, subside in many survivors, and persist in others in the form of chronic PTSD [5]. Accordingly, PTSD might be seen as a "disorder of recovery" from the early responses to psychologically traumatic events [5]. Chronic PTSD is highly reactive to environmental reminders of the traumatic event and to renewed life-stressors, and thus may have a fluctuating course [5]. In 1994, Rwanda endured one of the worst genocides of the 20^th^ century in which almost one million people perished in a period of 100 days [8]. Victims of the Genocide against Tutsis were slaughtered, raped, terrorized and maimed all over the country [9]. Death occurred by decapitation, clubbing, starvation and drowning among other methods. As a result, there was mass displacement of the population to the camps whereby large numbers of people died of illness, exhaustion and starvation [9]. To date, genocide survivors still suffer the consequences of the genocide [8-13]. Studies have shown the prevalence of PTSD to be as high as 30% in the general Rwandan population [10, 11, 13]. In a seven-day period in April every year, Rwandans gather to mourn the victims of the genocide against Tutsis. Testimonies, songs and documentary videos about the genocide are used to help people mourn by remembering their families and friends who perished during the genocide as a tool to build a violence free country in the future and repair broken hearts. However, during these commemoration activities, survivors living with chronic PTSD manifest symptoms. Most of them experience PTSD acute exacerbations that manifest as flashbacks, agitation, self-mutilation, avoidance, anger, fear, crying etc [8, 9]. While previous studies have assessed prevalence of, and factors associated with PTSD in Rwanda [10, 11, 13], data regarding PTSD acute exacerbations during commemoration periods remain scarce. Therefore, the objective of this study was to describe the magnitude of, and factors associated with severity of PTSD acute exacerbations during the annual commemoration periods of the 1994 genocide against Tutsis in Rwanda.

## Methods

**Study context**: Rwanda, a small and low-income country in East Africa, is divided into 30 administrative districts [14]. Until 4 years ago, the country had one medical school housed within the National University of Rwanda (currently known as University of Rwanda), which was located in Huye district in the Southern Province of Rwanda. In an effort to contribute to promotion of mental health in Rwanda, students at the National University of Rwanda (NUR) created the *Medical Students' Mental Health Association (MMHA)* in 2006. The association counts over 100 members, including medical, clinical psychology and social work students. In 2007, the MMHA started the *Mental Health Empowerment Project in Southern Province (MHEP)* that focused on psycho-education in neighboring schools, intervention for counseling during annual genocide commemoration periods and outreach activities to genocide survivors. Given the scarcity of mental health professionals, MMHA managed to train and avail students with enough skills to offer psychological interventions across all the 14 sectors of Huye District every year during the Genocide commemoration period.

**Data source**: This study used data retrieved from de-identifiable information collection forms used by MMHA members while intervening as counselors during the genocide commemoration week, 2011 (April 7^th^ to April 13^th^ 2011) in Huye District in the Southern Rwanda. Consistent with the American Psychiatric Association's Diagnostic and Statistical Manual of Mental Disorders, 4^th^ edition (DSM-IV), PTSD was defined as symptom clusters of re-experiencing, avoidance, negative alterations in cognition/mood, and alterations in arousal and reactivity [4]. We included people who had PTSD and experienced an acute exacerbation during the annual commemoration week of the 1994 genocide against Tutsis in 2011 in Huye District. It is estimated that 4363 people had trauma crisis across the country during that commemoration week in 2011 [8]. This study was approved by NUR's Faculty of Medicine and MMHA.

**Study design**: This study used a retrospective cross-sectional design involving secondary analysis of data collected primarily as part of the MMHA/MHEP project described earlier.

**Measures**: Our outcome measure included duration of PTSD acute exacerbations categorized into three levels: < 15 minutes, 15-30 minutes, and > 30 minutes. Traumatic attacks lasting for more than 30 minutes were considered as "severe" PTSD acute exacerbations. Drawing on previous research [2, 10, 13], we hypothesized that the following variables would potentially be associated with severe PTSD acute exacerbations: sex, age, number of children/ parents, presenting signs and information about frequency of previous PTSD acute attacks.

**Statistical analysis**: Descriptive analysis of the study sample was performed using frequencies and percentages. Bivariable and multivariable ordinal logistic regression models were fit to assess potential factors associated with severe (longer) PTSD acute exacerbations. We first performed a bivariable ordinal logistic regression analyses to assess the association between each potential factor and outcome measure (i.e PTSD acute exacerbations) to check which factors pass an initial screening, with a significance level set at 0.2. All potential factors independently associated with our outcome during the unadjusted analysis were retained for further assessment in the multivariable analysis. We selected the most parsimonious model using backward stepwise selection approach. We used "polr" command from the "MASS" package to fit the ordinal logistic regression models [15]. We reported associations as odds ratio (OR) and related 95% confidence interval (CI). Statistical analyses were two-tailed and p values of < 0.5 were considered to show statistical significance. All statistical analyses were performed using R software, version 3.3.1 [16].

## Results

The current study included 383 patients who presented with signs and symptoms of PTSD acute exacerbations during the genocide commemoration week (Table 1). The majority of patients were female (71.8%), aged 20-45years (53.5%), or had no parents (82.2%). Flashback and agitation were the predominant clinical pictures observed in 28.8% and 21.1%, respectively (Table 1). All participants reported history of PTSD acute exacerbations, of which 59.8% had experienced more than two PTSD acute exacerbations during the previous commemoration periods. 33.2% had severe PTSD acute exacerbations or exacerbations that lasted more than 30 minutes (Table 1). Having experienced more PTSD acute exacerbations in the past (OR = 1.86; 95% CI = 1.27-2.75) and having lost a partner in genocide (OR = 2.19; 95% CI=1.01-4.81) were associated with severe PTSD acute exacerbations (i.e. prolonged PTSD acute exacerbations that lasted more than 30 minutes), adjusting for sex and age (Table 2). Having children and / or parents were not significantly associated with severe PTSD acute exacerbations. Flashbacks were significantly associated with longer PTSD acute exacerbations in the unadjusted model (Table 2).

**Table 1:** Characteristics of the study participants (N=383)

		Duration of PTSD acute exacerbations, n (%)		
		< 15 minutes	15-30 minutes	> 30 minutes
**Characteristics**		91 (23.7)	165 (43.1)	127 (33.2)
**Sex**, n (%)				
Male	108 (28.2)	31 (28.7)	39 (36.1)	38 (35.2)
Female	275 (71.8)	60 (21.8)	126 (45.8)	89 (32.4)
**Age (years)**, n (%)				
<20	63 (16.4)	20 (31.7)	26 (41.3)	17 (26.9)
20-45	205 (53.5)	43 (20.9)	91 (44.4)	71 (34.6)
> 45	115 (30.0)	28 (24.3)	48 (41.7)	39 (33.9)
**Marital status**, n (%)				
Single	124 (32.4)	31 (25)	58 (46.8)	35 (28.2)
Married	114 (29.8)	27 (23.7)	49 (43.0)	38 (33.3)
Divorced	63 (16.4)	17 (26.9)	27 (42.9)	19 (30.2)
Widow (er)	82 (21.4)	16 (19.5)	31 (37.8)	35 (42.7)
**Have children**, n (%)				
No	201 (52.5)	46 (22.9)	91 (45.3)	64 (31.8)
Yes	182 (47.5)	45 (24.7)	74 (40.7)	63 (34.6)
**Have parent(s)**, n (%)				
No	315 (82.2)	75 (23.8)	134 (42.5)	106 (33.7)
Yes	68 (17.8)	16 (23.5)	31 (45.6)	21 (30.9)
**Prior PTSD acute exacerbations**, n (%)				
≤Twice	154 (40.2)	44 (28.6)	75 (48.7)	35 (22.7)
More than twice	229 (59.8)	47 (20.5)	90 (39.3)	92 (40.2)
**Clinical presentation**, n (%)				
Suicide ideation	42 (11.1)	15 (35.7)	16 (38.1)	11 (26.2)
Flashbacks	110 (28.7)	12 (10.9)	55 (50)	43 (39.1)
Stupor	51 (13.3)	16 (31.4)	23 (45.1)	12 (23.5)
Self-mutilation	66 (17.2)	18 (27.3)	23 (34.8)	25 (37.9)
Agitation	81 (21.1)	19 (23.5)	39 (48.1)	23 (28.4)
Other	33 (8.6)	11 (33.3)	9 (27.3)	13 (39.4)

PTSD, posttraumatic stress disorder

**Table 2:** Ordinal logistic regression analysis of factors associated with severe PTSD acute exacerbations

	PTSD acute exacerbations	
Characteristics	*Crude OR (95% CI)*	*Adjusted OR (95% CI)*
**Sex**		
Male	Reference	Reference
Female	1.09 (0.71-1.66)	1.07 (0.69-1.67)
**Age (years)**		
< 20	Reference	Reference
20-45	1.57 (0.93-2.68)	1.16 (0.61-2.20)
> 45	1.43 (0.80-2.55)	0.63 (0.27-1.44)
**Marital status**		
Single	Reference	Reference
Married	1.17 (0.73-1.88)	1.04 (0.59-1.81)
Divorced	1.00 (0.57-1.76)	0.94 (0.48-1.82)
Widow (er)	1.67 (1.00-2.84)	2.19 (1.01-4.81)
**Have children**		
No	Reference	
Yes	1.03 (071-1.50)	
**Have parent (s)**		
No	Reference	
Yes	0.94 (0.57-1.52)	
**Prior PTSD acute exacerbations**		
≤Twice	Reference	Reference
More than twice	1.90 (1.30-2.80)	1.86 (1.27-2.75)
**Presenting signs and symptoms**		
Suicide ideation	Reference	
Flashbacks	2.50 (1.28-4.92)	
Stupor	1.05 (0.48-2.27)	
Self- mutilation	1.70 (0.81-3.57)	
Agitation	1.44 (0.71-2.92)	
Other	1.52 (0.62-3.71)	

PTSD, posttraumatic stress disorder; OR, odds ratio; CI, confidence interval

## Discussion

The primary of objective of this study was to identify potential factors associated with severe PTSD acute exacerbations. We found that the odds of having severe PTSD acute exacerbations were greater among people with previous PTSD episodes (more than twice) and those who lost a spouse during the genocide. A history of acute PTSD attack in prior commemoration periods was associated with longer duration of symptoms during new attacks, suggesting that PTSD acute attacks tend to be more severe in people with chronic PTSD. Consistent with previous research that suggested that losing a partner to be a risk factor for PTSD [17], we found that having lost a partner during the genocide was associated with longer or severe PTSD acute exacerbations. This study found that females were more affected by PTSD acute exacerbations than males, and this difference may be explained by the number of females in the general population and the fact that many survivors are females and females are more likely to have experienced more traumatic antecedents of traumatic events like sexual abuse such as rape that may have led to unwanted pregnancies and/or HIV infection [18]. Hysteric crises could also have played a big role in this discrepancy of prevalence between females and males. Comparable results were obtained by Cohen et al (2009) who showed that independent predictors for increased depressive symptoms and PTSD included HIV infection and a history of genocidal rape [18]. As previously reported by Boscarino and Adams (2008), younger individuals were at higher risk of developing PTSD acute exacerbations [19]. The frequency of PTSD acute exacerbations among individuals aged less than 20 years was 16.4%. These are people who were not yet born during the genocide or were too young to witness the genocide atrocities given the current study used data collected in 2011 (i.e, 17 years after the genocide). This suggests the potential existence of secondary psychological trauma in our population [20]. This study showed that being an orphan was not significantly associated with developing longer or severe PTSD acute exacerbations. Similar findings reported by Schaal et al (2010) showed that the bereavement status did not impact the severity of prolonged grief reactions [21]. However, Munyandamutsa et al (2009) report that being orphan was associated with a higher risk of PTSD [12]. The current study has limitations that need to be acknowledged. First, given that we performed a secondary analysis on data that were not collected primarily for research, we were unable to include other important risk factors (e.g, injury, rape, substance abuse) and resilience factors (e.g, memberships in support group) for PTSD in our analysis [17]. Second, given that patients were asked to recall information related to frequency of previous PTSD acute exacerbations, recall bias cannot be ruled out. This recall bias might have led to under (over) reporting and misclassification of frequency of previous PTSD acute exacerbations. Lastly, this study only reports on PTSD acute exacerbations during the annual commemoration period of the genocide against Tutsis in Rwanda. As such, further studies should investigate the prevalence of and potential factors associated with PAE in other stressing situations such as social conflicts and domestic violence.

## Conclusion

PTSD acute exacerbation remains a major mental health challenge in the post-genocide Rwanda. People who reported having more prior PTSD acute exacerbations and being widow (er) were more likely to have severe PTSD acute exacerbations. Increasing access to mental healthcare services particularly during the commemoration periods should remain a priority. While history of PTSD acute exacerbations and bereavement status are non-modifiable factors, our findings could help identify and develop targeted interventions for these people who are at risk for severe PTSD acute exacerbations when they encounter triggering events.

### What is known about this topic

- Post-traumatic stress disorder commonly occurs after exposure to life threatening events. Rwanda endured a genocide whereby killings happened in communities;
- Most survivors witnessed brutal killings and others were directly victims of brutal assaults (physical and sexual);
- Prevalence of post-traumatic stress disorder has been reported to be as high as 30% in Rwanda.

### What this study adds

- Post-traumatic stress disorder acute exacerbation remains a major mental health challenge in the post-genocide Rwanda;
- People who reported having more prior post-traumatic stress disorder acute exacerbations and being widow (er) were more likely to have severe post-traumatic stress disorder acute exacerbations.

## Competing interests

The authors declare no competing interest.
